# Drug-Associated Vanishing Bile Duct Syndrome: Screening for Potential Pharmaceutical Triggers Using the WHO Pharmacovigilance Database

**DOI:** 10.3390/life16081232

**Published:** 2026-07-25

**Authors:** João Pereira Soares, Andreas E. Kremer, Jérôme Bonzon

**Affiliations:** 1 Department of Clinical Pharmacology and Toxicology, University Hospital Zurich, 8091 Zurich, Switzerland; joao.pereirasoares@usz.ch; 2 Faculty of Medicine, University of Zurich, 8006 Zurich, Switzerland; andreas.kremer@usz.ch; 3 Department of Gastroenterology and Hepatology, University Hospital Zurich, 8091 Zurich, Switzerland

**Keywords:** vanishing bile duct syndrome, drug-induced liver injury, pharmacovigilance, drug safety, hepatotoxicity, adverse drug event

## Abstract

Vanishing bile duct syndrome (VBDS) is a rare cholestatic liver disease often associated with drug-induced liver injury, yet systematic data on pharmaceutical triggers remain limited. Using WHO VigiBase, we applied Bayesian disproportionality analysis (IC_0.25_) to identify drug-event associations that may not be readily apparent in clinical trials or pre-marketing studies. Product labels approved by Swissmedic or the FDA, as well as LiverTox, were reviewed to determine whether VBDS was already acknowledged as an adverse event. Signal detection was deliberately restricted to reports naming a single suspect drug. Among these single-agent reports, 22 drugs demonstrated a positive IC_0.25_ signal, of which nevirapine, dapsone and azithromycin showed the strongest disproportionality signal. Only one of these agents (carbamazepine) explicitly labelled VBDS as an adverse event. These findings are based on spontaneous reporting data: disproportionality analysis is a hypothesis-generating signal-detection method that does not establish causality and requires further validation. This study expands the current understanding of drug-induced VBDS by reinforcing the associations with known drugs and generating pharmacovigilance signals for new potential VBDS triggers across several drug categories.

## 1. Introduction

First described in 1988, the vanishing bile duct syndrome (VBDS) is a rare condition characterized by a progressive destruction and disappearance of the intrahepatic bile ducts, resulting in bile duct loss (ductopenia) and cholestasis [1]. Throughout this article, the syndrome is referred to exclusively as vanishing bile duct syndrome, although the terms drug-induced cholangiopathy and bile duct injury are also encountered in the literature. Several causes and triggers have been associated with ductopenia, including congenital and genetic diseases, autoimmune conditions such as acute and chronic liver rejection, sarcoidosis and small-duct primary sclerosing cholangitis, as well as neoplasms, infections and xenobiotics [1,2]. The exact incidence and prevalence of drug-induced VBDS are not known. The US Drug-Induced Liver Injury Network (DILIN) reported in 2017 bile duct loss of varying degrees in 26 of 363 (7%) DILI patients who underwent liver biopsy. Antibiotics like amoxicillin/clavulanic acid were among the most frequent causes of DILI in this cohort [3].

Drug-induced VBDS is predominantly a clinical and pathological diagnosis [2]. The diagnosis of drug-induced VBDS, and particularly its milder forms, relies therefore heavily on expertise in hepatic pathology and requires the exclusion of other causes of ductopenia. Liver biopsy plays a pivotal role in the diagnosis. Histologically, VBDS is defined as the loss of at least half of the interlobular ducts in a biopsy with at least 10 portal areas or a bile duct to portal tract ratio of <0.5 [3]. Histological lesions can be found in the biliary epithelium of interlobular ducts located between the cholangioles (or ductules) and second or third generation septal bile ducts, which, interestingly, are also the main targets in other immune-mediated diseases, such as graft-versus-host disease, primary biliary cholangitis or small-duct primary sclerosing cholangitis [4]. 

The clinical presentation is heterogeneous [2], but typically occurs after an episode of severe cholestatic hepatitis, often with immunoallergic features, such as rash, fever, facial oedema, lymphadenopathy and eosinophilia, and in more severe cases with Stevens–Johnson syndrome or toxic epidermal necrolysis [2]. The presence of hypersensitivity features or other extrahepatic features is variable. A latency period of generally 1 to 6 months after the onset of drug-related liver injury has been described [2]. In some instances, the acute onset can be delayed until after the discontinuation of the drug [4].

Formally, for the diagnosis of drug-induced VBDS, the following conditions must be met: 1. Persistent elevations in serum alkaline phosphatase and bilirubin for more than 6 months after onset of drug-induced liver injury; 2. Absence of clinical or serological evidence of primary biliary cholangitis, primary or secondary sclerosing cholangitis and graft-versus-host disease; 3. Liver biopsy findings of paucity of intralobular bile ducts in a sample taken at least 1 month after onset of injury [2].

Hepatotoxicity patterns that solely consist of cholestasis are relatively rare events; mixed-type liver injury is far more common; however, at presentation in acute stages, bile duct damage may or may not be present [4]. The outcome varies: while in most cases the clinical and histological picture is largely reversible, some patients may progress towards liver failure and require liver transplantation [3]. Due to the low incidence reported in the literature, a standardized treatment of VBDS has not been established; hence, this rare condition should be managed at a tertiary liver referral centre [1].

Drug-induced bile duct injury is considered a hepatic manifestation of a T cell-mediated hypersensitivity reaction against the administered xenobiotic. This concept is supported by the aforementioned eosinophilia, history of allergy, possible concomitant Stevens–Johnson syndrome or toxic epidermal necrolysis, but also by shortening of the latency period with repeated exposure to the drug and lymphocyte sensitization [3]. The inflammatory response, which is primarily directed against cholangiocytes, is usually associated with prolonged cholestasis and ultimately leads to bile duct degeneration and loss [3]. However, the precise biliary damage mechanisms in drug-induced VBDS remain largely unknown [5]. Among immunological biliary diseases, it is generally accepted that the detection of allo- or autoantigens on biliary epithelial cells by immune cells triggers a sequence of events that ultimately leads to biliary injury, where CD3-positive T cells generally predominate (the dominant T cell (CD4+ or CD8+) varies with disease state). In the presence of an appropriate costimulatory signal, proinflammatory cytokines are released, leading to proliferation of cytotoxic T lymphocytes and further to more antigen presentation and recruitment of immune cells [6]. Combined with T-cell cytotoxicity, the cell apoptosis leads to bile duct injury and ductopenia. Irreversible ductopenia occurs when apoptosis exceeds the proliferative response [6]. The cell death receptor CD95 (Fas) and its ligand (FasL), perforin and granzyme B, tumour necrosis factor-alpha, oxidative stress leading to DNA damage, and down-regulation of Bcl-2 have all been implicated in bile duct injury [6].

Substances that have been reported in several case studies as causes of VBDS can include the following: beta-lactams (penicillins, cephalosporins), fluoroquinolones, sulphonamides, macrolides, atypical and typical antipsychotics, tricyclic antidepressants, non-steroidal anti-inflammatory drugs, proton pump inhibitors and herbal compounds (such as glycyrrhizin, artemisinin, tibolone), among others [1,2,3]. 

Over the past few years, a few disproportionality analyses have begun to explore drug-associated VBDS using the US Food and Drug Administration Adverse Event Reporting System (FAERS). Wang et al. identified thirteen antibacterial agents with significant VBDS reporting associations, with fluoroquinolones showing the highest positive signals, and, in a companion analysis, reported four non-steroidal anti-inflammatory drugs (notably ibuprofen, with loxoprofen showing the highest signal value) as associated with VBDS [7,8]. More recently, Karkra et al. reported a broader FAERS analysis of drug-induced VBDS [9], and Wei et al. characterised biliary disorders associated with immune checkpoint inhibitors in FAERS [10]. Although valuable, these analyses draw predominantly on a single, largely US-based reporting system and, individually, focus on selected pharmacological classes rather than on the full spectrum of implicated drugs.

The WHO Global Pharmacovigilance Database, VigiBase, aggregates more than 35 million anonymized individual case safety reports of suspected adverse effects of medicines and vaccines since 1978 and contributed by over 180 member countries of the WHO Programme for International Drug Monitoring [11], and therefore offers substantially broader geographic, demographic and prescribing-pattern coverage than FAERS. This wider coverage is particularly advantageous for a rare adverse drug reaction such as VBDS, for which single national systems accrue few cases, and it reduces the dominance of country-specific reporting behaviour. 

Against this background, a clearly definable knowledge gap remains: no disproportionality analysis has yet examined drug-associated VBDS across the full VigiBase dataset, nor systematically triangulated the resulting signals against three independent regulatory and reference sources (LiverTox, FDA and Swissmedic labelling). The present study addresses this gap, complements and cross-validates the existing FAERS-based literature, and aims to characterize the full range of drugs reported in association with VBDS using the WHO Global Pharmacovigilance Database VigiBase. 

## 2. Materials and Methods

A search using VigiLyze (an online signal detection and signal management tool), closely integrated with VigiBase (WHO Pharmacovigilance Global Database [11]), compiled all the individual case safety reports (ICSRs) related with VBDS. Due to the anonymized data from an open-access database, authorization from the Swiss ethics committee was not required. 

To assess if VBDS was a known/reported adverse event, the original drug/product label of each drug, either approved by Swissmedic (the Swiss authority responsible for the authorisation and supervision of therapeutic products) or by the FDA (Food and Drug Administration, the authority responsible for the authorisation and supervision of therapeutic products in the United States of America), was consulted in November 2024, looking for “vanishing bile syndrome” and “verschwindende Gallengänge Syndrom” or the related histological finding “ductopenia” and “Duktopenie”. Additionally, Livertox, a central repository of clinical information focused on the prevention and control of drug-induced liver injury, was consulted. Livertox is a joint effort of the Liver Disease Research Branch of the National Institute of Diabetes and Digestive and Kidney Diseases (NIDDK) and the National Library of Medicine (NLM), National Institutes of Health, that provides up-to-date, comprehensive, and unbiased information about drug-induced liver injury.

**Figure 1:**
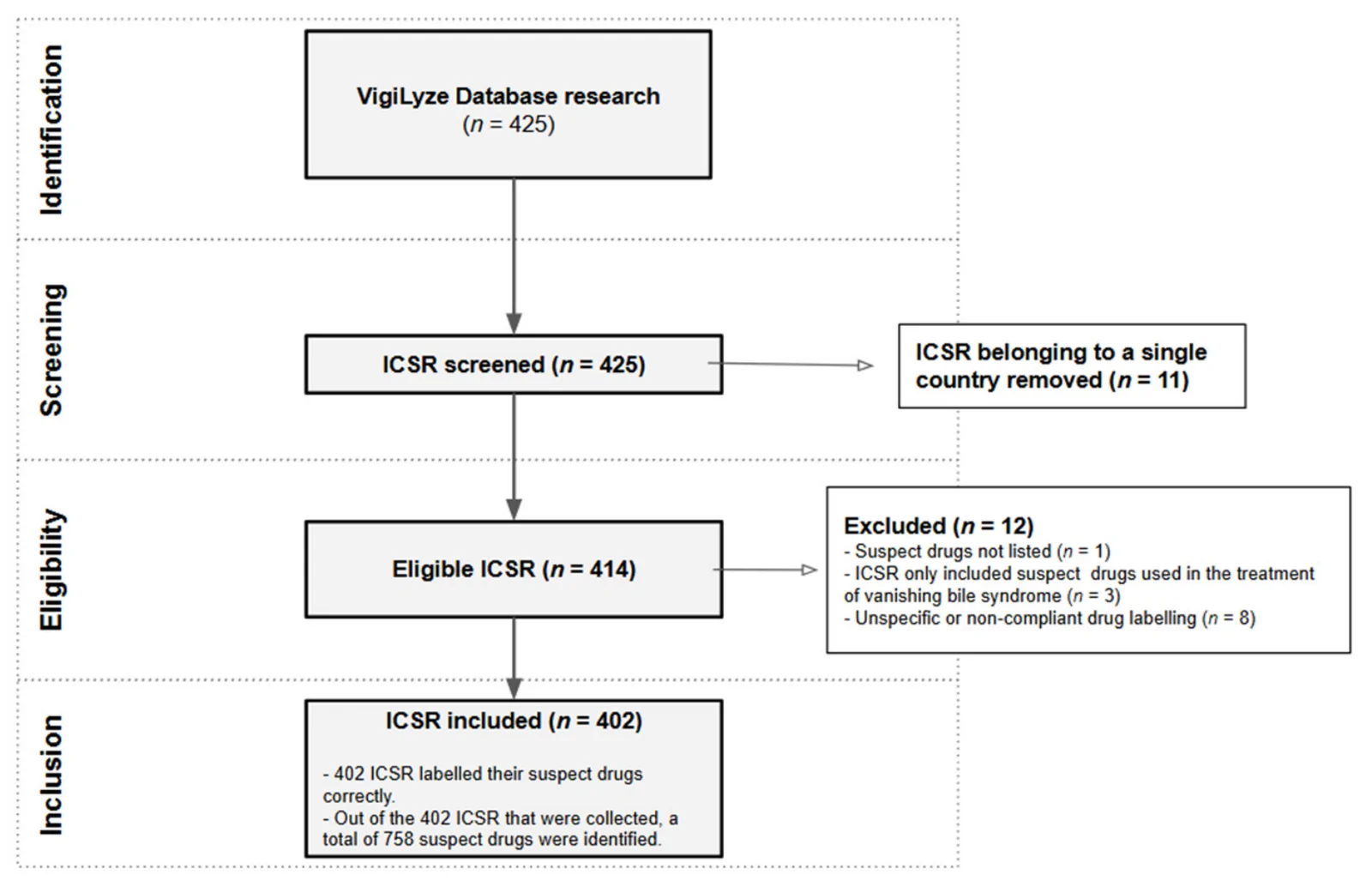
Methodology of the study.

Duplicate detection and follow-up management were handled within VigiBase/VigiLyze prior to extraction. VigiBase applies algorithms to flag suspected duplicate reports, and successive follow-up versions of a report are consolidated so that each ICSR is represented once by its most recent (latest) version. Only deduplicated records were exported. Each exported ICSR was subsequently reviewed manually for internal consistency (drug role, active-ingredient coding according to the WHO Drug Dictionary) before signal detection, and records that did not meet the inclusion criteria were removed as detailed above and in Figure 1.

### 2.1. Inclusion and Exclusion Criteria

Inclusion Criteria:

- Anonymized and deduplicated individual case safety reports (ICSRs) in VigiBase using VigiLyze, which were associated with the MedDRA System Organ Classes (version 27.1) preferred term (PT) “vanishing bile duct syndrome” (which compiled the Lowest Level Terms (LLTs) “vanishing bile duct syndrome” and “ductopenia”), since 17 October 2005 (first case reported) until 5 November 2024 (extraction date). No separate high-level terms, high-level group terms or Standardised MedDRA Queries (SMQs) were applied, as VBDS is represented as a MedDRA PT.
- Only drugs labelled in the ICSR as “suspect”.

Exclusion Criteria:

- Single ICSRs originating from a single country, in order to reduce country-specific reporting bias and/or healthcare-system-related confounding.
- Reports on drugs used in the treatment of VBDS or similar conditions, such as primary biliary cholangitis (including ursodeoxycholic acid, obeticholic acid or corticosteroids (dexamethasone, prednisone, budesonide, prednisolone, methylprednisolone)), to minimize bias. An ICSR was still considered valid if, apart from these substances, other drugs were also listed as suspect.
- Drugs labelled as “NOS” (not otherwise specified) or without explicit labelling of the active ingredient according to the WHO Drug Dictionary.
- Drugs whose role was labelled as “interacting” or “concomitant”.

### 2.2. Signal Detection and Disproportionality Analysis

In this study, the case-non-case analysis was conducted for each drug within the extracted dataset from the pharmacovigilance database. The Information Component (IC) was calculated using the Bayesian Confidence Propagation Neural Network (BCPNN) method developed by the Uppsala Monitoring Centre (UMC) [12,13]. Specifically, the method compares the proportion of a given adverse event associated with a drug to the proportion of the same adverse event for all other treatments in the WHO Pharmacovigilance Database. It is conceptually defined as: IC = log_2_ [P(D,E)/(P(D) × P(E))], where P(D,E) represents the probability of the drug–event combination, and P(D) and P(E) represent the marginal probabilities of the drug and event, respectively [14]. Although IC is known for its conservatism when compared to the reporting odds ratio (ROR), IC mitigates the risk of false-positive signals and increases the reliability of the results [13]. It is important to emphasise that the case-non-case (disproportionality) analysis employed in this study is a signal detection tool and does not constitute, nor is it intended to constitute, a formal causality assessment of VBDS. Potential clinically relevant signals warrant further investigation with other study designs.

IC_0.25_ is the lower limit of the 95% credibility interval for the IC. When a positive IC_0.25_ (IC_0.25_ > 0) is identified, it is considered that a statistical association between a drug and the adverse event is present [12,13]. A positive IC_0.25_ suggests that there is a higher likelihood that the observed association is not due to random chance. This single threshold of IC_0.25_ > 0 was used consistently throughout this study. IC_0.25_ was captured through the VigiLyze interface. 

The descriptive demographic and clinical characterisation was based on the complete set of 402 valid ICSRs. For signal detection, however, and to reduce the risk of polytherapy-related bias, only ICSRs that referenced a single “suspect” drug and yielded a positive signal (IC_0.25_ > 0) were retained for the disproportionality ranking. Drug combinations were excluded from this ranking, as the IC is vulnerable to polytherapy bias and cannot reliably indicate which co-administered drug is truly associated with the event. Examples of combinations found include amoxicillin/clavulanic acid, sulfamethoxazole/trimethoprim, lamivudine/zidovudine, emtricitabine/tenofovir, doravirine/lamivudine/tenofovir and efavirenz/emtricitabine/tenofovir. In addition, all reports referring to “tenofovir” were judged incomplete and prone to bias. Because two formulations are currently marketed (tenofovir disoproxil and tenofovir alafenamide), and due to insufficient information to distinguish between them, reports containing “tenofovir”, “tenofovir disoproxil” or “tenofovir alafenamide” were excluded from the single-agent signal analysis.

### 2.3. Statistics and Management of Missing Data

Data was collected from VigiLyze^®^ and recorded in a Microsoft Excel spreadsheet (MS 2016, Microsoft Corp., Redmond, WA, USA). Descriptive analysis on demographic and clinical characteristics of reported cases was provided. Statistical analyses were performed in IBM SPSS Statistics for Windows Version 29.0 (IBM Corp, Armonk, NY, USA). The majority of the ICSRs included more than one suspected drug. Each ICSR was analysed to assess severity. According to the WHO definition, severity was classified in the following categories: death, life-threatening, caused prolonged hospitalization, disabling/incapacitating, congenital/birth defect and other medically important conditions. To each drug or drug combination, the IC_0.25_ was calculated and captured through the VigiLyze interface.

The data was not complete for every ICSR in the following categories: sex, age, time to onset, severity, performed de-challenge and reporter qualification. A comprehensive analysis was solely conducted on the data that was accessible. The heterogeneity and frequent absence of basic information such as sex, age, time to onset, and reporter qualification, as well as information on individual risk factors such as comorbidities, polypharmacy, and allergy history, limited the possibility of systematic analysis. Including such incomplete data falls outside the primary scope of this study, which is pharmacovigilance signal detection, and would risk introducing selection bias and reducing the robustness of the findings. Partially reported findings (for example, time-to-onset, documented in only 65 of 402 ICSRs) are therefore presented as descriptive, exploratory observations rather than as extensive population-level estimates.

## 3. Results

### 3.1. Drug Selection

A total of 425 ICSRs of VBDS were initially identified, spanning from October 2005 (first report) to November 2024 (database extraction date). After applying the inclusion and exclusion criteria, 23 ICSRs were excluded from the final analysis: 11 cases corresponded to a single report from one country, 1 case only considered “interacting” and “concomitant” drugs, 8 cases failed to explicitly mention the active ingredient of the suspected drug according to the WHODrug (either labelled as “uncoded” or reported as “Vitamins NOS”, “Penicillins NOS”, “Ayurvedic preparation NOS”, “Vitamin E NOS”) and 3 cases labelled “ursodeoxycholic acid”, “obeticholic acid” and/or the aforementioned corticosteroids as the only suspect drug. Only 402 ICSRs were analysed further (Figure 1), from which a total of 758 reports for drug suspects were recognized, corresponding to 213 different drugs/drug combinations.

### 3.2. Demographic and Clinical Characteristics

Demographic and clinical characteristics are summarised in Table 1. 

**Figure 2:**
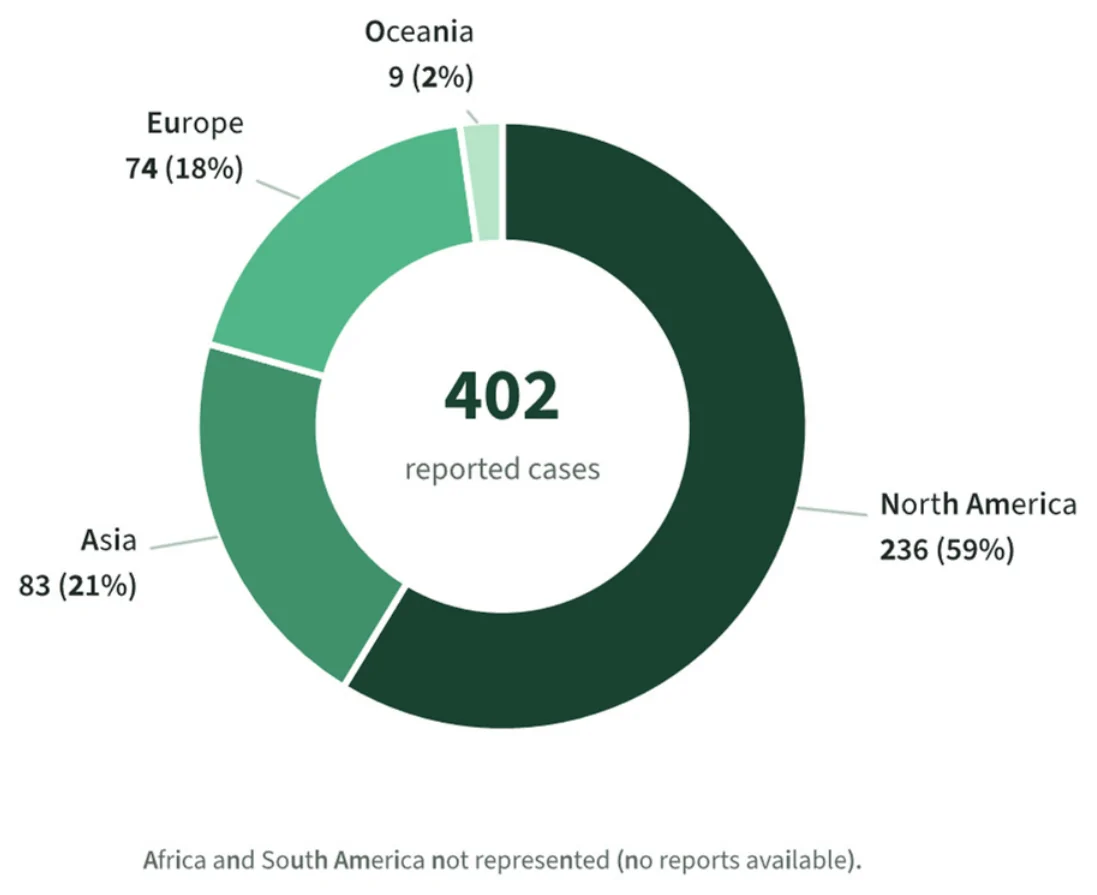
Distribution of the reported cases by continent (Africa and South America are not represented).

Most cases were male (*n* = 180). Of the total 402 cases, 96.8% were classified as serious. The mean age was 44.3 years (SD 23.5; range: 4 to 85 years). Most cases were reported from North America (led by the United States of America, *n* = 235 cases), followed by Europe (headed by the United Kingdom, *n* = 18) and Asia (represented by Japan, *n* = 72, and India, *n* = 7) (Figure 2). 

The VBDS occurred after a mean time to onset of 38.3 days (maximal range: 0–390 days, data available from 65 cases). As seen in Table 2, most cases occurred within 8–31 days (61.5%), followed by 0–7 days (21.5%), 1–3 months (10.8%), 3–6 months (4.6%), and >6 months (1.5%). Early-onset cases were mainly associated with antibacterials and anti-inflammatory/antirheumatic products, whereas the 8–31-day interval was dominated by antibacterials, antineoplastic agents, and antiepileptics (Table 2).

**Figure 3:**
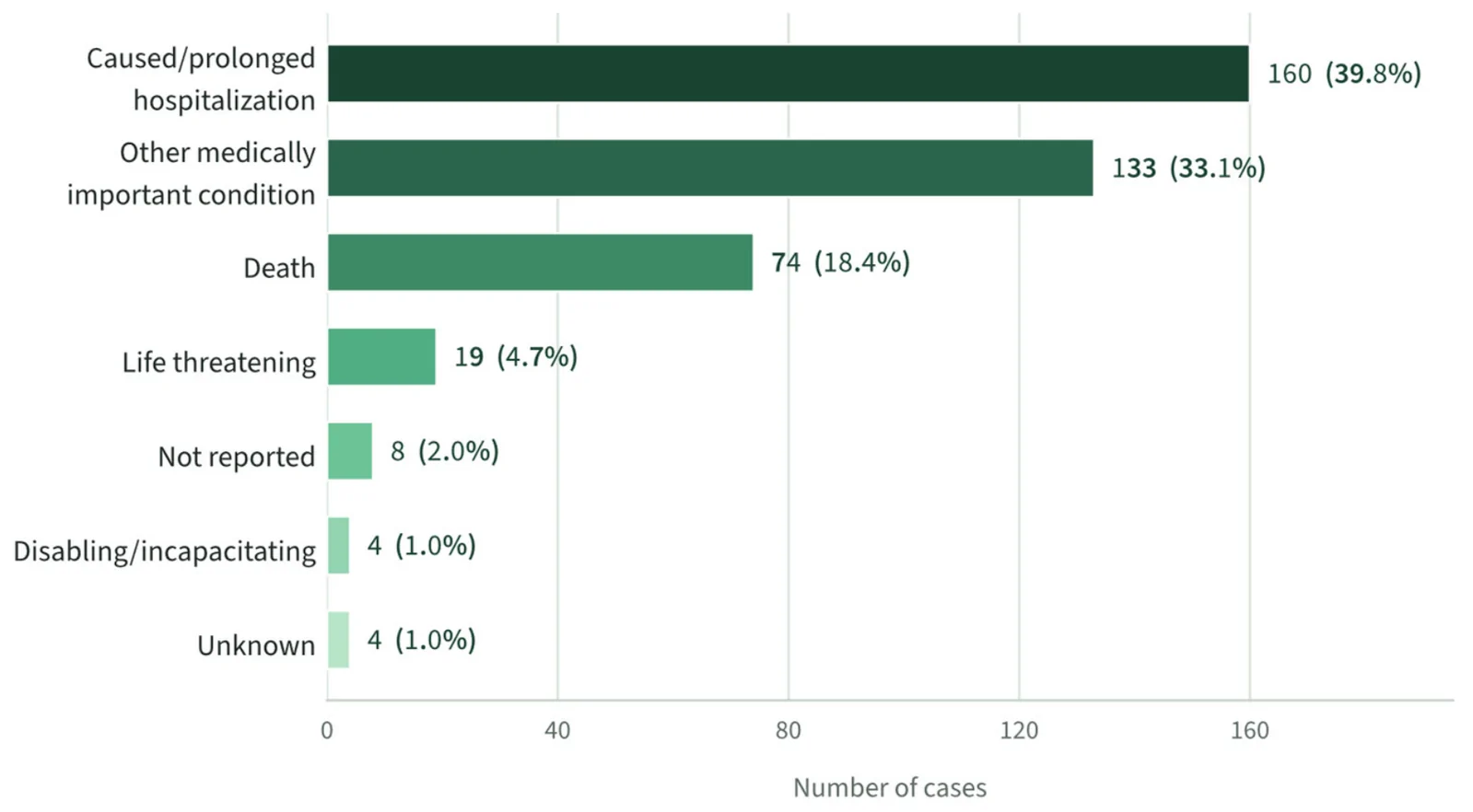
Distribution of cases by seriousness criteria.

Later-onset cases were uncommon and were distributed across a small number of drug classes (based on the Anatomical Therapeutic Chemical (ATC) Classification). The most commonly reported outcome was “caused/prolonged hospitalization”, followed by “other medically important condition” (Figure 3). 

Severity differed across pharmacological classes. The highest proportions of fatal reports were observed for direct-acting antivirals (64.8%) and antimycobacterials (68.4%), whereas antibacterials, the most frequently reported class, showed a lower fatal proportion (24.6%) and were mainly associated with hospitalization (Supplementary Table S4). Antineoplastic agents, immunosuppressants, and antiepileptics were more commonly classified as other medically important conditions. Liver transplantation or graft-related events were explicitly coded in 9 of the 402 ICSRs (2.2%): liver transplant (*n* = 4), liver transplant rejection (*n* = 4) and complications of a transplanted liver (*n* = 1). Because transplantation is not systematically captured in spontaneous reports, this figure represents a minimum estimate, and the true proportion progressing to transplantation cannot be determined from these data. Liver histology was not systematically captured in spontaneous reports: in the present dataset, none contained sufficient detail to confirm ductopenia against the criteria mentioned in the Introduction.

**Figure 4:**
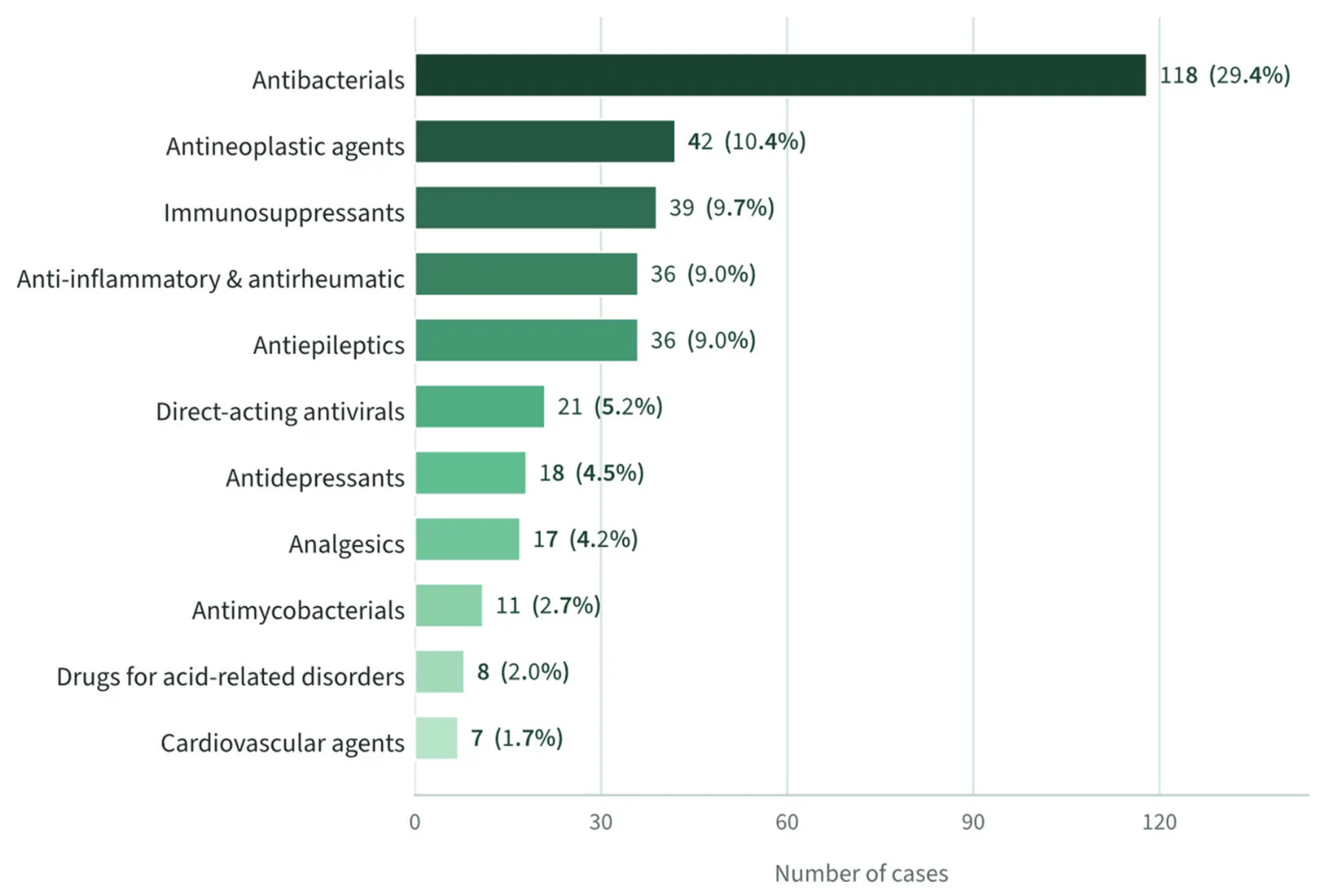
Distribution of the most reported suspect drugs in the 10 most common ATC-based drug categories (*n* = 402; only 353 ICSRs are represented. The missing ICSRs fall into categories that are not represented in this distribution). The vast majority of the suspect drugs reported from the gathered ICSRs (*n* = 402) correspond to antibacterial agents (for example, amoxicillin, amoxicillin/clavulanic acid, azithromycin, levofloxacin, meropenem, ceftriaxone, etc.), followed by antineoplastic agents, such as bevacizumab, bortezomib, busulfan, ipilimumab, gemcitabine, etc., and immunosuppressant drugs, such as ciclosporin, azathioprine, infliximab, mercaptopurine, and others.

The most reported suspected drugs were divided into the following ATC-guided categories: antibacterials, antineoplastic agents, immunosuppressants, analgesics, anti-inflammatory and anti-rheumatic drugs, antiepileptics, and antimycobacterials (other categories are also listed in Supplementary Table S3). Figure 4 clearly illustrates the overproportioned representation of the category of antibacterials.

### 3.3. Drug Characteristics

All different drug reports (including single drugs and drug combinations) were compiled, and only those with a statistically relevant signal (IC_0.25_ > 0) were subsequently analysed (*n* = 68, 63 individual drugs and 5 fixed-dose combinations; Supplementary Table S1). Because the majority of ICSRs listed more than one suspect drug, and to minimise confounding by co-administered agents, the ranking analyses below were restricted to the subset of case reports naming a single suspect drug, corresponding to 22 individual agents with a positive IC_0.25_ (Supplementary Table S2). The complete quantitative signal list for all drugs and drug combinations with a positive IC_0.25_ (IC_0.25_ > 0), including the number of observed ICSRs, the IC_0.25_ lower bound, the ATC-based class and the LiverTox, FDA and Swissmedic labelling status for VBDS, is provided in Supplementary Table S1 so that readers can independently appraise the relative strength of each individual signal.

Levofloxacin (*n* = 19, IC_0.25_ = 2.2), sertraline (*n* = 15, IC_0.25_ = 2.6), ibuprofen (*n* = 11, IC_0.25_ = 2.2), lamotrigine (*n* = 10, IC_0.25_ = 2.9) and infliximab and paracetamol (*n* = 8, IC_0.25_ = 2.9, 2.7 and 2.6 respectively) were the most reported single drugs with IC_0.25_ > 0. The drugs with the highest IC_0.25_ originating from single drug reports are (in descending order): nevirapine (*n* = 2, IC_0.25_ = 3.7), dapsone (*n* = 21, IC_0.25_ = 3.5), azithromycin (*n* = 3, IC_0.25_ = 3.1), lamotrigine (*n* = 10, IC_0.25_ = 16) and flucloxacillin (*n* = 7, IC_0.25_ = 2.8) (Supplementary Table S2).

We explicitly distinguish reporting frequency (the absolute number of observed ICSRs for a drug, which is driven largely by prescription volume and reporting behaviour) from signal strength (the IC_0.25_, which quantifies how disproportionate that reporting is relative to the rest of the database). The two do not necessarily coincide: levofloxacin was among the most frequently reported drugs yet had a moderate IC_0.25_ (2.2), whereas nevirapine and dapsone were reported far less often but produced the strongest disproportionality signals (IC_0.25_ = 3.7 and 3.5). 

For the bibliographic review, only reports with a single suspect drug were considered. Of the 22 single agents with a statistically significant disproportionality signal (IC_0.25_ > 0), 7 were not referenced as possible causes of drug-induced VBDS in the FDA or Swissmedic labelling or in LiverTox: levofloxacin, infliximab, minocycline, dapsone, mercaptopurine, bortezomib, and carbocisteine. Only one agent, carbamazepine, was labelled as being associated with VBDS by both Swissmedic and the FDA. The following search terms were used in the information provided by both Swiss and US health authorities: “vanishing bile syndrome”, “ductopenia”, ”verschwindende Gallengänge Syndrom” and “Duktopenie”. Interestingly, LiverTox mentions other agents that have been reported as drug culprits for VBDS but express a negative IC_0.25_, such as lenalidomide, thalidomide, hydrochlorothiazide, phenobarbital, ezetimibe, allopurinol, oxcarbazepine, and fenofibrate.

## 4. Discussion

The results of this study highlight the significant and varied pharmacological landscape of drugs associated with VBDS. By consulting the WHO Global Pharmacovigilance Database, we identified 68 positive disproportionality signals (63 individual drugs and 5 fixed-dose combinations) with a potential statistically significant association with VBDS. To reduce polytherapy-related confounding, the ranking and bibliographic analyses focused on the 22 individual agents identified from reports naming a single suspect drug. This provides a crucial step towards understanding potential pharmaceutical triggers and possibly anticipating or preventing the clinically relevant progression of this rare and often severe hepatic condition.

Spontaneous reporting systems are inherently vulnerable to reporting bias and confounding by indication: drugs used in severe, multisystem disease (for example, oncology, autoimmune or transplant settings) may be preferentially reported, and underlying conditions or concomitant therapies can both mimic and precipitate cholestatic injury, complicating causal attribution [15]. Additional biases specific to spontaneous-reporting systems warrant explicit consideration. Notoriety bias means that drugs already known to be hepatotoxic may attract disproportionate reporting of any liver-related event, including VBDS, particularly after safety communications or publications, thereby inflating their apparent signal. Protopathic bias may arise when a drug is prescribed for an early, still-undiagnosed manifestation of the very biliary or cholestatic disease that is later reported as the adverse event, producing an incorrect association. Indication bias and differences in prescription volume act in the same direction. These mechanisms should temper the interpretation of both established and newly emerging signals as they are most likely to affect high-volume, high-notoriety drug classes and are least able to explain signals for rarely reported agents. They cannot be quantified within VigiBase^®^ and are further debated against our findings. 

Among the single-agent reports, the drugs most frequently associated with VBDS were levofloxacin, sertraline, ibuprofen, lamotrigine, infliximab and paracetamol. These drugs span various therapeutic categories, including antibiotics, analgesics, antiepileptics, and immunosuppressants, suggesting that VBDS-associated pharmacovigilance signals emerge across a broad range of pharmacological classes. Proposed mechanisms of hepatobiliary injury in the literature vary by drug class. Considered together, these mechanistic considerations are not invoked as proof of causality but as a biologically plausible interpretive framework for the disproportionality signals presented above. As outlined in the Introduction, drug-induced bile duct injury is regarded as a hepatic manifestation of a T cell-mediated hypersensitivity reaction directed against cholangiocytes [3,6], with irreversible ductopenia occurring when cholangiocyte apoptosis exceeds the proliferative response. The drug classes that dominate the IC_0.25_ ranking in this study are precisely those previously reported in the same literature as triggers of VBDS: fluoroquinolones, macrolides, NSAIDs, antiepileptics and beta-lactams have all been described as causative agents in case series and reviews of drug-induced VBDS [1,2,3], and the immunoallergic phenotype of cholestatic hepatitis and the typical latency of one to six months were described for these classes [2]. 

As previously stated, the identification of a pharmacovigilance signal does not imply a causal relationship [12,13]. The observed associations reflect disproportionate reporting within a spontaneous reporting database and require confirmation through dedicated pharmacoepidemiologic studies. Irrespective of the precise pathophysiological mechanisms involved, systematic pharmacovigilance signal detection provides a valuable and complementary approach to identifying potential pharmaceutical triggers of VBDS that may not yet be fully captured in clinical trials or post-marketing surveillance [12,13,15]. When restricting the ranking to single-agent reports, the highest IC_0.25_ values were observed for nevirapine, dapsone, azithromycin and lamotrigine. Nevirapine, an antiretroviral used in the treatment of HIV [16], produced the strongest single-agent signal, underscoring the importance of vigilant hepatic monitoring in patients receiving long-term antiretroviral therapy, although a direct causal relationship with VBDS cannot be established from this dataset alone. Dapsone, an antibiotic used for leprosy and Pneumocystis jirovecii pneumonia prophylaxis [17,18], also emerged with a high IC_0.25_, highlighting a potential risk in patients with chronic infections or those receiving prophylactic treatments pending further epidemiological confirmation. 

Other HIV-related drugs generating positive signals, including antiretroviral medications such as lamivudine, atazanavir or ritonavir, as well as dapsone, raise a specific concern of confounding by indication. In people living with HIV, the underlying infection, opportunistic co-infections (including viral hepatitis B and C), immune reconstitution and immune-mediated cholangiopathy can each cause cholestatic bile-duct disease independently of any drug. The same reasoning applies to dapsone. The signals for these agents may therefore partly reflect the clinical context in which they are prescribed rather than an intrinsic drug effect, and this indication-related confounding cannot be adjusted for within a spontaneous-reporting dataset. 

The demographic analysis revealed that most cases of VBDS were reported from North America, with the United States accounting for the majority of reports. This is most likely attributable to structural factors such as more established regulatory and pharmacovigilance frameworks, higher spontaneous-reporting rates, broader access to healthcare and newer therapeutic standards, and greater use of certain implicated drugs in these settings, rather than reflecting a true excess incidence of VBDS in North America. Reporting cultures and legal frameworks, healthcare system infrastructure, resources available for detecting and confirming drug-induced hepatic injury and drug utilization patterns vary substantially across regions, and the present findings may not be directly generalizable to countries with different reporting practices. The possibility that reporting biases, including stimulated reporting following media attention or regulatory actions, may have contributed to the observed geographic distribution cannot be excluded.

The interpretation of these geographic differences, therefore, requires caution and is best framed as a map of reporting activity rather than of disease incidence. Mature national systems (for example, the United States, the United Kingdom and Japan, which dominated our dataset) combine high reporting rates, well-resourced diagnostic pathways, including access to liver biopsy, and large prescribing volumes for the implicated classes. Conversely, the near-absence of reports from large regions with substantial antiretroviral and antimicrobial exposure most plausibly reflects under-reporting and limited pharmacovigilance infrastructure rather than a genuinely lower risk. Regional differences in the drugs in use (such as the antiretroviral and antimycobacterial regimens prominent in some settings) further shape which signals emerge. These considerations reinforce that VigiBase^®^, despite its breadth, reflects the structure of the contributing systems and cannot be read as an epidemiological incidence estimate.

Although the WHO pharmacovigilance database has been operational since 1978 [13], the first report in this analysis dates from 2005. Possibly due to the rarity of this syndrome, the awareness of this condition in the medical community may be lower and not as widespread worldwide, resulting in a low number of reports. This further supports the likelihood that this syndrome is largely underdiagnosed and underreported. A further limitation of this analysis is the absence of systematic information on histological assessment in the majority of ICSRs. 

The predominance of severe cases (96.8% classified as serious) and the significant mortality rate (18.4%) highlight the clinical importance of timely diagnosis and intervention. The additional class-based severity analysis further showed that severity was not evenly distributed across pharmacological classes. Among the major classes, direct-acting antivirals and antimycobacterials showed the highest proportions of fatal reports, whereas antibacterials, although the most frequently reported class overall, were more commonly associated with hospitalization than with fatal outcomes. Antineoplastic agents, immunosuppressants and antiepileptics were more often characterised by “other medically important condition” as the predominant seriousness criterion. These findings add granularity to the overall severity profile and suggest that VBDS-associated reports may differ not only in frequency but also in clinical seriousness depending on the pharmacological context in which they occur.

The clinical significance of these class-based differences should nonetheless be interpreted cautiously, as they are as likely to reflect reporting and contextual factors as intrinsic drug toxicity. The high fatal proportions for direct-acting antivirals and antimycobacterials probably reflect the severity of the underlying conditions (advanced HIV, disseminated mycobacterial infection) and frequent multi-organ involvement, so that death may be misattributed to VBDS when it is driven by the underlying disease; fatality coding in ICSRs captures the case outcome rather than an event specifically caused by ductopenia. The predominance of hospitalization among antibacterial reports is consistent with their very high reporting volume and generally acute, reversible presentations, whereas the “other medically important condition” pattern for antineoplastic and immunosuppressant agents fits the chronic, closely monitored oncology and transplant settings in which they are used. Residual confounding by indication and co-medication cannot be excluded.

The variability in the time to onset of VBDS, with a mean of 38.3 days but a wide range from 0 to 390 days, suggests that the syndrome can develop insidiously over a long period or acutely shortly after drug exposure. When the time to onset was stratified into clinically relevant intervals, most cases occurred within 8 to 31 days, followed by 0 to 7 days, whereas later-onset cases beyond 3 months were uncommon. Early-onset cases were mainly associated with antibacterials and anti-inflammatory/antirheumatic products, while the 8- to 31-day interval was dominated by antibacterials, antineoplastic agents and antiepileptics. This temporal distribution is clinically relevant because it suggests that the risk window for VBDS reporting is concentrated in the first month after exposure for many implicated classes, although delayed presentations remain possible and should not be overlooked. This latency variability is well recognised in the literature on drug-induced bile duct injury and poses a challenge for clinicians seeking to attribute drug exposure to subsequent hepatobiliary changes, particularly in patients receiving multiple potentially offending agents [19,20].

These temporal patterns are pharmacologically coherent, although they rest on only 65 informative reports and must be interpreted as exploratory. The short latency seen with antibacterials and NSAIDs is consistent with an idiosyncratic, immune-mediated (hypersensitivity) mechanism in a previously sensitised host, in which re-exposure or a brief treatment course precipitates cholestatic injury within days to a few weeks (mirroring the classical 1-to-6-week window described for immunoallergic drug-induced cholestasis). Intermediate latencies for antineoplastic agents, immunosuppressants and antiepileptics are compatible with cumulative-dose or metabolism-dependent injury and with the delayed, T cell–driven cholangiocyte damage characteristic of aromatic anticonvulsants. The rare late-onset cases (beyond three months, e.g., lipid-modifying agents and antidepressants) may reflect slowly progressive, insidious ductopenia or delayed recognition. Clinically, this implies that the reporting risk window is concentrated in the first month after exposure for most implicated classes, but that delayed presentations occur and should not be overlooked, particularly in patients on multiple potentially offending agents [20]. Because the time to onset was available for so few cases, these associations are hypothesis-generating and require confirmation in datasets with more complete chronological information.

Placing our findings alongside the recent FAERS-based literature provides a degree of external cross-validation while also revealing informative discrepancies. Concordant signals include the antibacterial classes highlighted by Wang et al.—fluoroquinolones (levofloxacin, ciprofloxacin), macrolides (azithromycin, clarithromycin), beta-lactams and trimethoprim/sulfamethoxazole—all of which also produced positive IC_0.25_ signals in VigiBase [7], and the NSAID signal dominated by ibuprofen and loxoprofen reported by the same group [8], both of which appear among our positive signals. The broad drug-induced VBDS pattern described by Karkra et al. in FAERS is likewise reproduced here [9], and the immune checkpoint inhibitor–associated biliary disorders characterised by Wei et al. correspond to the pembrolizumab and nivolumab signals identified in our dataset [10] (ipilimumab, also implicated by Wei et al., was reported in our dataset but did not reach a positive IC_0.25_). Discordant or additional signals in VigiBase include a prominent cluster of antiretroviral agents (nevirapine, lamivudine, atazanavir, ritonavir) and immunosuppressants (tacrolimus, ciclosporin, mycophenolate, antithymocyte immunoglobulin, basiliximab, infliximab) that are less conspicuous in the US-centric FAERS analyses, plausibly reflecting VigiBase’s broader international reach and differing regional prescribing and disease epidemiology. This convergence on well-established culprits, together with divergence driven by geographic coverage, supports the complementary value of a VigiBase-based approach rather than treating the two data sources as interchangeable.

The emergence of signals for newer drugs such as pembrolizumab, bortezomib, and other targeted or immunomodulatory agents may partly reflect market growth, access to differentiated health systems, changing prescribing patterns (including broadening of therapy indications) or improved awareness of immune-related cholangiopathies [21,22,23], in addition to the possibility of a true drug-related risk. This underscores the need for longitudinal pharmacoepidemiologic studies to discriminate between increased use and genuine novel hepatotoxicity signals as potential genuine risk that has not yet been fully captured in traditional reference sources. These new signals could help healthcare professionals include VBDS in the differential diagnosis of unexplained cholestatic liver injury in patients exposed to these agents, whilst recognising that confirmatory studies are required before any causal inference can be drawn.

Interestingly, among the assessed 22 single agents with a positive signal, VBDS was labelled for only one drug (carbamazepine), by both Swissmedic and the FDA. Furthermore, 7 of these single agents (levofloxacin, infliximab, minocycline, dapsone, mercaptopurine, bortezomib and carbocisteine) were not previously referenced in major databases like LiverTox, Swissmedic, or the FDA. 

The divergence between our signals and current labelling should be read in the light of the different objectives and evidence thresholds governing the two processes. Pharmacovigilance signal detection is intentionally sensitive and hypothesis-generating: a positive IC_0.25_ flags a disproportionately reported association that merits further scrutiny, at the deliberate cost of specificity. Regulatory labelling, by contrast, results from a confirmatory, benefit–risk exercise that generally incorporates a new adverse reaction only after causality has been supported by converging evidence (well-documented individual cases with positive dechallenge/rechallenge, biological plausibility, epidemiological data and, where available, controlled studies). A drug can therefore legitimately generate a strong VigiBase signal for VBDS while remaining absent from its label, without either source being in error. The discrepancies we report should be interpreted as prompts for regulatory re-evaluation rather than as evidence of labelling incompleteness.

From a regulatory perspective, the divergence between the pharmacovigilance signals identified in this study and the information currently provided in product labels by the FDA, Swissmedic, and LiverTox [2,24,25] suggests that a reassessment of existing labelling may be warranted for selected drugs. Active post-marketing surveillance initiatives (such as targeted pharmacoepidemiologic studies or product label updates following regulatory review) could help close this gap and improve patient safety.

From a clinical standpoint, these findings suggest that clinicians prescribing such agents should maintain a heightened index of suspicion for cholestatic liver injury, incorporate appropriate hepatic monitoring into follow-up protocols, and report suspected cases to national pharmacovigilance systems to further characterise these signals.

Translating these observations into concrete recommendations, two points can be made. First, when prescribing the drugs that generated a signal without existing VBDS recognition, clinicians should not withhold clinically indicated therapy but should adopt heightened pharmacovigilance: baseline and periodic liver-function testing (with particular attention to a cholestatic pattern), prompt investigation of unexplained cholestasis, early drug withdrawal when VBDS is suspected, and referral to a hepatology centre. Second, we recommend that suspected VBDS cases involving the newly flagged drugs be reported to national pharmacovigilance centres and, through them, to the WHO Uppsala Monitoring Centre, so that continued accrual of high-quality, well-documented ICSRs can strengthen or refute these preliminary signals. 

The study limitations, inherent to its retrospective nature, include potential underreporting and the absence of formal causality assessment [19]. The incompleteness of data across several variables (including sex, age, time to onset, and reporter qualification) is also acknowledged as a limitation inherent to spontaneous reporting systems. It is important to emphasise that the case-non-case (disproportionality) analysis used in this study is a signal detection methodology and was not designed to, nor does it, establish a causal relationship between any specific drug and VBDS [13]. Causality assessment instruments such as the Roussel Uclaf Causality Assessment Method (RUCAM), the Maria and Victorino scale, or other related algorithms are specifically designed for comprehensive clinical evaluation of individual, well-documented DILI cases [26,27]. Because pharmacovigilance datasets rely on ICSRs, which often lack the detailed clinical and chronological information required for these structured assessment tools, their application is not methodologically appropriate in studies based on large spontaneous reporting systems. Applying causality tools to incomplete ICSR data would introduce systematic misclassification and selection bias, thereby undermining the statistical validity of the analysis. Moreover, it is important to highlight that the study’s findings deliver clinically informative pattern recognition within the reporting dataset, but they do not establish independent causal toxicity risks for individual drug classes. In particular, fatality coding in ICSRs may reflect incomplete outcome capture, and residual confounding by underlying disease, indication, co-medication and reporting practice remains likely.

A limitation of fundamental importance, and central to the interpretation of every signal reported here, is the near-complete absence of histological confirmation. Liver histology is not systematically captured in spontaneous reports: in the present dataset, a liver biopsy was mentioned in only 3 of the 402 ICSRs, none with sufficient detail to confirm ductopenia. The great majority of cases, therefore, represent reporter-assigned VBDS diagnoses, and the proportion meeting the full clinicopathological criteria cannot be verified. It is worth noting that histological confirmation of each reported case lies ultimately within a formal causality assessment and is outside the scope of a pharmacovigilance analysis. This constraint is shared by the recent FAERS-based analyses [7,8,9,10], which likewise identified cases through spontaneous reports coded by MedDRA preferred terms but did not confirm diagnosis with information on biopsy-confirmed ductopenia. Furthermore, none of these studies explicitly addressed the absence of histological confirmation as a limitation, underscoring both the methodological commonality across spontaneous-reporting studies of VBDS and the greater diagnostic transparency adopted here.

Overall, these limitations highlight the need for further prospective studies and pharmacoepidemiologic investigations, including Post-Authorisation Safety Studies (PASS), initiated by sponsors or at the request of regulatory agencies, to validate these findings, elucidate the underlying mechanisms, and, where appropriate, establish a causal relationship between drug exposure and VBDS. Future studies should specifically assess whether the temporal clustering within the first month after exposure, the heterogeneity in severity across ATC classes and the association with increasing numbers of suspect drugs can be confirmed in designs less vulnerable to reporting bias. Such evidence would also inform regulatory decision-making, including product label updates and direct healthcare professional communications [28].

## 5. Conclusions

This study expands the current understanding of drug-induced VBDS by reinforcing statistical associations with known drugs and identifying new potential pharmacovigilance signals across several drug categories. Among the single-agent reports analysed, levofloxacin, sertraline, ibuprofen and lamotrigine were the most frequently reported drugs, while nevirapine, dapsone, azithromycin and lamotrigine demonstrated the strongest disproportionality signals. Only carbamazepine explicitly mentions VBDS in its product label. Because causality cannot be inferred from disproportionality data, these results should be regarded as signal detection rather than confirmation of drug-specific risk. Given the severity and potential irreversibility of VBDS, early recognition and withdrawal of the suspected offending drug remain critical. Further pharmacoepidemiologic evaluation of the seven newly identified unlabelled single agents (levofloxacin, infliximab, minocycline, dapsone, mercaptopurine, bortezomib and carbocisteine) is warranted. Such evidence would further support clinical, therapeutic and regulatory decision-making, including, for instance, early and appropriate disease management and issuance of direct healthcare professional communications or the update of product labels.

## Figures and Tables

**Table 1:** Baseline characteristics of the study population.

Characteristic	*n* = 402 (100%)
Female, *n* (%)	162 (40.3%)
Male, *n* (%)	180 (44.8%)
Age, years (mean ± SD [min, max])	44.3 ± 23.5 [4, 85]
Age group, *n* (%)	
Child (0–12 years)	46 (11.4%)
Adolescent (13–17 years)	22 (5.5%)
Adult (18–64 years)	186 (46.3%)
Elderly (>65 years)	90 (22.4%)
Unknown	58 (14.4%)
Continent, *n* (%)	
Asia	83 (20.6%)
Oceania	9 (2.2%)
Europe	74 (18.4%)
North America	236 (58.7%)
Time to onset, days (mean [min, max]) (*n* = 65)	38.3 [0, 390]
Number of suspect drugs (mean [min, max])	2.4 [1, 31]
Serious, *n* (%)	
Yes	389 (96.8%)
No	8 (2.0%)
Unknown	3 (0.7%)
Seriousness criteria, *n* (%)	
Caused/prolonged hospitalization	160 (39.8%)
Disabling/incapacitating	4 (1.0%)
Life threatening	19 (4.7%)
Death	74 (18.4%)
Other medically important condition	133 (33.1%)
Not reported	8 (2.0%)
Dechallenge performed?/Reaction resolved?, *n* (%)	
Yes/Yes	80 (19.9%)
Yes/No	32 (8.0%)
Yes/Unknown	51 (12.7%)
Unknown/Yes	42 (10.4%)
Unknown/No	43 (10.7%)
Unknown/Unknown	87 (21.6%)
Not reported	62 (15.4%)

**Table 2:** Time-to-onset intervals and predominant drug classes.

Time-to-Onset Interval	Cases, *n* (%)	Predominant ATC-Based Classes
0–7 days	14 (21.5%)	Antibacterials; anti-inflammatory/antirheumatic products
8–31 days	40 (61.5%)	Antibacterials; antineoplastic agents; antiepileptics
1–3 months	7 (10.8%)	Antibacterials; immunosuppressants; direct-acting antivirals
3–6 months	3 (4.6%)	Lipid-modifying agents; antimycotics
>6 months	1 (1.5%)	Antidepressants

## Data Availability

The data that support the findings of this study are available from the corresponding author upon reasonable request.

## Supplementary Materials

The following supporting information can be downloaded at https://www.mdpi.com/article/10.3390/life16081232/s1, Table S1: List of drugs, according to number of reports of the curated dataset (in descending order). ranked in descending order. LiverTox® listing status: Present = drug is referenced in LiverTox as a cause of, or associated with, VBDS; Absent = not referenced. FDA/Swissmedic label status: Present = vanishing bile duct syndrome (or associated terms) is mentioned in the product label; Absent = not mentioned); Table S2: List of drugs from ICSRs with just a single suspect drug (in descending order by number of reports, presented only those with IC_0.25_ > 0); Table S3: Categories of drugs of the reported cases (in descending order); Table S4: Categories of drugs of the reported cases (in descending order). Percentages are calculated within each pharmacological class (denominator = number of suspect-drug reports for that class). “Death” corresponds to reports flagged as fatal. Only the classes discussed in the text are shown; additional classes and less frequent seriousness criteria (life-threatening, disabling/incapacitating) are not tabulated. Overall, 96.8% of the 402 ICSRs were classified as serious and 18.4% were fatal.

## Abbreviations

The following abbreviations are used in this manuscript:

ATC	Anatomical Therapeutic Chemical
BCPNN	Bayesian Confidence Propagation Neural Network
CIOMS	Council for International Organizations of Medical Sciences
DILI	Drug-induced liver injury
FAERS	FDA Adverse Event Reporting System
FDA	U.S. Food and Drug Administration
ICSR	Individual Case Safety Report
PASS	Post-Authorisation Safety Study
RUCAM	Roussel Uclaf Causality Assessment Method
UMC	Uppsala Monitoring Center
VBDS	Vanishing bile duct syndrome
WHO	World Health Organization
