# Supplementary material for: Drug-Associated Vanishing Bile Duct Syndrome: Screening for Potential Pharmaceutical Triggers Using the WHO Pharmacovigilance Database
Source: Life (Basel). 2026 Jul 25;16(8):1232. doi: 10.3390/life16081232 (PMC13514335; doi:10.3390/life16081232)
Supplement: Supplementary file 1 [file life-16-01232-s001.zip › life-4431853-supplementary.pdf]

Table S1. List of drugs, according to number of reports of the curated dataset (in descending order). ranked in descending order. LiverTox® listing status: Present = drug is referenced in LiverTox as a cause of, or associated with, VBDS; Absent = not referenced. FDA/Swissmedic label status: Present = vanishing bile duct syndrome (or associated terms) is mentioned in the product label; Absent = not mentioned).

WHODrug active ingredient	Number of reports	%	$IC_{0.25}$	Label FDA/ Swissmedic	Reference in LiverTox
Sulfamethoxazole, Trimethoprim	30	4.0	3.3	Present[1]	Present
Paracetamol	27	3.6	2.6	Absent	Present
Azithromycin	21	2.8	3.1	Absent	Present
Levofloxacin	20	2.6	2.2	Absent	Absent
Ibuprofen	19	2.5	2.2	Absent	Present
Nevirapine	17	2.2	3.7	Absent	Present
Lamotrigine	16	2.1	2.9	Absent	Present
Celecoxib	16	2.1	2.7	Absent	Present
Tacrolimus	16	2.1	2.6	Absent	Absent
Sertraline	15	2.0	2.6	Absent	Present
Ciprofloxacin	12	1.6	1.6	Absent	Present
Lamivudine	12	1.6	3.1	Absent	Absent
Dapsone	12	1.6	3.5	Absent	Absent
Infliximab	11	1.5	1.1	Absent	Absent
Pembrolizumab	11	1.5	2.4	Absent	Present
Atazanavir	10	1.3	3.1	Absent	Absent
Tenofovir	10	1.3	3.1	Absent	Absent
Ritonavir	10	1.3	2.9	Absent	Absent
Clarithromycin	10	1.3	2.2	Absent	Present
Meropenem	10	1.3	2.5	Absent	Present
Flucloxacillin	9	1.2	2.8	Absent	Present
Minocycline	9	1.2	2.7	Absent	Absent
Atorvastatin	9	1.2	0.8	Absent	Present
Carbamazepine	9	1.2	1.7	Present[2]	Present
Cyclosporine	9	1.2	2.0	Absent	Absent
Temozolomide	7	0.9	2.1	Absent	Present
Zidovudine	7	0.9	2.0	Absent	Absent
Antithymocyte immunoglobulin	7	0.9	2.0	Absent	Absent
Amoxicillin, Clavulanic acid	7	0.9	0.2	Absent	Present
Nivolumab	7	0.9	1.0	Absent	Present
Omeprazole	7	0.9	0.6	Absent	Present
Ceftriaxone	6	0.8	-	-	-
Amoxicillin	6	0.8	0.4	Absent	Present
Cefalexin	6	0.8	1.6	Absent	Present
Didanosine	6	0.8	2.1	Absent	Absent

Doxycycline	6	0.8	1.3	Absent	Absent
Zonisamide	5	0.7	1.8	Absent	Present
Hydrochlorothiazide, Triamterene	5	0.7	1.7	Absent	Yes[3]
Fluconazole	5	0.7	1.2	Absent	Absent
Levetiracetam	5	0.7	1.1	Absent	Absent
Mycophenolate mofetil	5	0.7	2.0	Absent	Absent
Mycophenolate sodium	5	0.7	2.0	Absent	Absent
Bortezomib	5	0.7	0.9	Absent	Absent
Azathioprine	4	0.5	0.6	Absent	Present
Metronidazole	4	0.5	-	-	-
Lenalidomide	4	0.5	-	-	Present
Moxifloxacin	4	0.5	1.1	Absent	Present
Lansoprazole	4	0.5	-	-	-
Tenofovir disoproxil	4	0.5	0.7	Absent	Absent
Erythromycin	4	0.5	-	-	-
Pexidartinib	4	0.5	1.4	Absent	Present
Valproate sodium	4	0.5	1	Absent	Present
Basiliximab	4	0.5	1.3	Absent	Absent
Carbocisteine	4	0.5	1.3	Absent	Absent
Cefepime	4	0.5	0.3	Absent	Absent
Diclofenac	4	0.5	-	-	-
Efavirenz	4	0.5	0.7	Absent	Absent
Emtricitabine, Tenofovir disoproxil	4	0.5	-	-	-
Etravirine	4	0.5	1.4	Absent	Absent
Methotrexate	4	0.5	-	-	-
Phentermine	4	0.5	0.9	Absent	Absent
Raltegravir	4	0.5	1.2	Absent	Absent
Vancomycin	4	0.5	-	-	-
Mercaptopurine	3	0.4	0.4	Absent	Absent
Ceftazidime	3	0.4	-	-	-
Cefdinir	3	0.4	0.6	Absent	Present
Emtricitabine	3	.4	0.7	Absent	Absent
Enfuvirtide	3	.4	0.7	Absent	Absent
Ethinylestradiol, Norgestimate	3	.4	0.5	Absent	Present
Montelukast	3	.4	0.0	-	Present
Busulfan	3	.4	0.5	Absent	Absent
Estradiol	3	.4	-	-	-
Estrogens conjugated	3	.4	-	-	Yes[4]
Thiotepa	3	.4	0.6	Absent	Absent
Ethambutol	3	.4	-	-	-
Cabozantinib	3	.4	0.7	Absent	Absent

Clopidogrel	3	.4	-	-	-
Fludarabine	3	.4	0.3	-	-
Gemcitabine	3	.4	-	-	-
Ipilimumab	3	.4	1.2	Absent	Absent
Lopinavir, Ritonavir	3	.4	0.4	Absent	Absent
Loxoprofen	3	.4	0.1	Absent	Absent
Rituximab	3	.4	-	-	-
Terbinafine	3	.4	-	-	-
Topiramate	3	.4	-	-	-
Pantoprazole	2	.3	-	-	-
Levocetirizine	2	.3	-	-	-
Lamivudine, Zidovudine	2	.3	-	-	-
Isoniazid	2	.3	-	-	-
Citalopram	2	.3	-	-	-
Caspofungin	2	.3	-	-	-
Hydrochlorothiazide	2	.3	-	-	Present
Phenobarbital	2	.3	-	-	Present
Valproic acid	2	.3	1	Absent	Present
Cefcapene	2	.3	-	-	-
Ethinylestradiol, Norethisterone	2	.3	-	-	-
Minoxidil	2	.3	-	-	Yes[4]
Thioridazine	2	.3	-	-	-
Cefazolin	2	.3	-	-	-
Paclitaxel	2	.3	-	-	-
Thiamazole	2	.3	-	-	-
Acetylsalicylic acid	2	.3	-	-	-
Apalutamide	2	.3	-	-	-
Cyclophosphamide	2	.3	-	-	-
Escitalopram	2	.3	-	-	-
Ezetimibe	2	.3	-	-	Present
Immunoglobulin human normal	2	.3	-	-	-
Levothyroxine	2	.3	-	-	-
Pazopanib	2	.3	0.1	-	-
Propylthiouracil	2	.3	-	-	-
Rifampicin	2	.3	-	-	-
Sulfamethoxazole	1	.1	-	-	Present
Nalfurafine	1	.1	-	-	-
Sulfasalazine	1	.1	-	-	-
Secukinumab	1	.1	-	-	-
Cilastatin, Imipenem	1	.1	-	-	-
Dapagliflozin, Metformin	1	.1	-	-	-

Indium (111 In) ibritumomab tiuxetan	1	.1	-	-	-
Pemafibrate	1	.1	-	-	-
Artemether, Lumefantrine	1	.1	-	-	-
Rifaximin	1	.1	-	-	-
Capecitabine	1	.1	-	-	-
Chorionic gonadotrophin	1	.1	-	-	-
Filgrastim	1	.1	-	-	-
Anastrozole	1	.1	-	-	-
Nitrofurantoin	1	.1	-	-	-
Olanzapine	1	.1	-	-	-
Teicoplanin	1	.1	-	-	-
Yttrium (90Y) ibritumomab tiuxetan	1	.1	-	-	-
Allopurinol	1	.1	-	-	Present
Diclofenac, Paracetamol	1	.1	-	-	-
Doravirine, Lamivudine, Tenofovir disoproxil fumarate	1	.1	-	-	-
Bevacizumab	1	.1	-	-	-
Atropa bella-donna, Caffeine, Dihydrocodeine phosphate, Diphenylpyraline hydrochloride, Guaifenesin, Methylephedrine hydrochloride, Noscapine hydrochloride hydrate, Paracetamol	1	.1	-	-	-
Carbimazole	1	.1	-	-	-
Dextropropoxyphene	1	.1	-	-	-
Edoxaban	1	.1	-	-	-
Mesalazine	1	.1	-	-	-
Metoclopramide	1	.1	-	-	-
Pertuzumab	1	.1	-	-	-
Quetiapine	1	.1	-	-	-
Valganciclovir	1	.1	-	-	-
Brentuximab vedotin	1	.1	-	-	-
Cefotaxime	1	.1	-	-	-
Clindamycin	1	.1	-	-	-
Efavirenz, Emtricitabine, Tenofovir disoproxil	1	.1	-	-	-
Enalapril	1	.1	-	-	-
Esomeprazole	1	.1	-	-	-
Foscarnet	1	.1	-	-	-
Insulin degludec	1	.1	-	-	-
Loperamide	1	.1	-	-	-
Lorazepam	1	.1	-	-	-

Melphalan	1	.1	-	-	-
Abacavir	1	.1	-	-	-
Carvedilol	1	.1	-	-	-
Clotrimazole	1	.1	-	-	-
Everolimus	1	.1	-	-	-
Hydralazine	1	.1	-	-	-
Imatinib	1	.1	-	-	-
Iron	1	.1	-	-	-
Oxcarbazepine	1	.1	-	-	Present
Plerixafor	1	.1	-	-	-
Regorafenib	1	.1	-	-	-
Thalidomide	1	.1	-	-	Present
Acetylsalicylic acid, Caffeine, Codeine	1	.1	-	-	-
Albendazole	1	.1	-	-	-
Alendronate	1	.1	-	-	-
Ampicilline, Sulbactam	1	.1	-	-	-
Arbekacin	1	.1	-	-	-
Aripiprazole	1	.1	-	-	-
Atovaquone	1	.1	-	-	-
Atovaquone, Proguanil	1	.1	-	-	Present
Benazepril	1	.1	-	-	-
Betamipron, Panipenem	1	.1	-	-	-
Carfilzomib	1	.1	-	-	-
Cefiderocol	1	.1	-	-	-
Cemiplimab	1	.1	-	-	-
Chlorphenamine maleate. Dihydrocodeine phosphate. Methylephedrine hydrochloride-dl	1	.1	-	-	-
Dabrafenib	1	.1	-	-	-
Durvalumab	1	.1	-	-	-
Ebastine	1	.1	-	-	-
Fenofibrate	1	.1	-	-	Present
Ferrous sulfate	1	.1	-	-	-
Fexofenadine	1	.1	-	-	-
Folic acid	1	.1	-	-	-
Goserelin	1	.1	-	-	-
Linezolid	1	.1	-	-	-
Medroxyprogesterone	1	.1	-	-	-
Meloxicam	1	.1	-	-	-
Mycophenolic acid	1	.1	-	-	-
Norethisterone	1	.1	-	-	-

Ofloxacin	1	.1	-	-	-
Piperacillin, Tazobactam	1	.1	-	-	-
Pitavastatin	1	.1	-	-	-
Platinum	1	.1	-	-	-
Prunus serotina bark	1	.1	-	-	-
Ramipril	1	.1	-	-	-
Rivaroxaban	1	.1	-	-	-
Roxadustat	1	.1	-	-	-
Ruxolitinib	1	.1	-	-	-
Silodosin	1	.1	-	-	-
Silybum marianum	1	.1	-	-	-
Simvastatin	1	.1	-	-	-
Sitagliptin	1	.1	-	-	-
Spironolactone	1	.1	-	-	-
Tozinameran	1	.1	-	-	-
Trametinib	1	.1	-	-	-
Trastuzumab	1	.1	-	-	-
Trastuzumab emtansine	1	.1	-	-	-
Triamterene	1	.1	-	-	-
Venlafaxine	1	.1	-	-	-
Vincristine	1	.1	-	-	-
Vonoprazan	1	.1	-	-	-

1 Referenced in Swissmedic
2 Referenced in both Swissmedic und FDA Label
3 Only Hydrochlorothiazide is referenced
4 Estrogens (not further specified) is listed in LiverTox.

Table S2. List of drugs from ICSRs with just a single suspect drug (in descending order by number of reports, presented only those with $IC_{0.25} > 0$)

WHODrug active ingredient variant	Number of reports	%	$IC_{0.25}$
Levofloxacin	19	10.0%	2.2
Sertraline	15	7.9%	2.6
Ibuprofen	11	5.8%	2.2
Lamotrigine	10	5.3%	2.9
Infliximab	8	4.2%	1.3
Paracetamol	8	4.2%	2.6
Flucloxacillin	7	3.7%	2.8
Minocycline	7	3.7%	2.7
Carbamazepine	6	3.2%	1.7
Pembrolizumab	6	3.2%	2.4
Meropenem	5	2.6%	2.5
Ciprofloxacin	4	2.1%	1.6
Amoxicillin	3	1.6%	0.4
Azithromycin	3	1.6%	3.1
Clarithromycin	3	1.6%	2.2
Dapsone	3	1.6%	3.5
Mercaptopurine	2	1.1%	0.4
Nevirapine	2	1.1%	3.7
Azathioprine	1	0.5%	0.6
Bortezomib	1	0.5%	0.9
Carbocisteine	1	0.5%	1.3
Valproate sodium	1	0.5%	1.0

Table S3. Categories of drugs of the reported cases (in descending order).

Categories	Frequency	Percentage
Antibacterials	118	29.4
Antineoplastic agents	42	10.4
Immunosuppressants	39	9.7
Anti-inflammatory and antirheumatic	36	9.0
Antiepileptics	36	9.0
Direct acting antivirals	21	5.2
Antidepressants	18	4.5
Analgesics	17	4.2
Antimycobacterials	11	2.7
Drugs for acid related disorders	8	2.0
Cardiovascular agents	7	1.7
Cough and cold preparations	5	1.2
Lipid modifying agents	5	1.2

Antihistamines	4	1.0
Antipsychotics	4	1.0
Antithrombotic agents	4	1.0
Sex hormones and modulators	4	1.0
Others with percentage < 1.0 %	23	5.8

Table S4. Categories of drugs of the reported cases (in descending order). Percentages are calculated within each pharmacological class (denominator = number of suspect-drug reports for that class). "Death" corresponds to reports flagged as fatal. Only the classes discussed in the text are shown; additional classes and less frequent seriousness criteria (life-threatening, disabling/incapacitating) are not tabulated. Overall, 96.8% of the 402 ICSRs were classified as serious and 18.4% were fatal.

Pharmacological class	Suspect-drug reports, n	Death, n (%)	Caused / prolonged hospitalisation, n (%)	Other medically important condition,	Predominant seriousness criterion
Antibacterials	195	48 (24.6)	85 (43.6)	43 (22.1)	Hospitalisation
Direct-acting antivirals	108	70 (64.8)	24 (22.2)	14 (13.0)	Death
Antimycobacterials	19	13 (68.4)	2 (10.5)	3 (15.8)	Death
Antineoplastic agents	86	17 (19.8)	25 (29.1)	27 (31.4)	Other medically important condition
Immunosuppressants	74	11 (14.9)	15 (20.3)	48 (64.9)	Other medically important condition
Antiepileptics	47	11 (23.4)	13 (27.7)	20 (42.6)	Other medically important condition
